# Sixth World Congress for Hair Research Cairns-On the Great Barrier Reef, Australia, 16-19 June, 2010

**DOI:** 10.4103/0974-7753.58549

**Published:** 2009

**Authors:** Rod Sinclair, Les Jones, Nick Rufaut, Allan Nixon

**Affiliations:** Department of Dermatology, St Vincent's Hospital, Melbourne, Australia. Email: leslie.jones@svhm.org.au


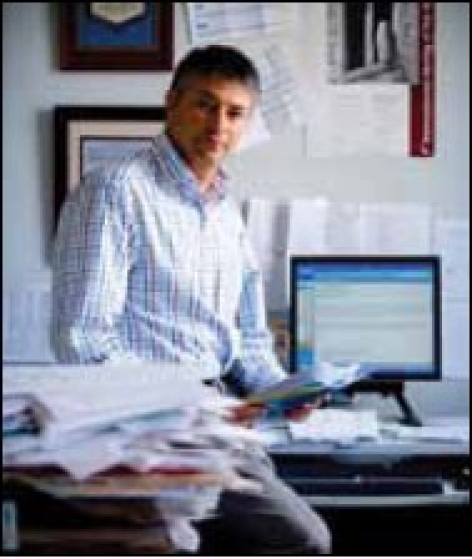


The 6^th^ World Congress of Hair Research is the premier event on the international hair research calendar. It will bring together hair biologists, wool and hair fiber scientists, clinical dermatologists and hair transplant surgeons in their united pursuit for increased knowledge of hair in health and disease. We welcome all delegates to this special event in the vibrant tropical city of Cairns, Australia, from the 16^th^ to the 19^th^ of June, 2010.

An exciting program has been organized, including Nobel Laureate Professor Peter Doherty (Physiology or Medicine 1996), Professor Ralf Paus, Professor George Cotsarelis, Professor Rod Sinclair, Dr. Yutaka Shimomura and Professor Seong-Jin Kim. Clinical symposia and specialized workshops preceding the main conference on Wednesday, the 16^th^ of June, will attract and merge both scientists and clinicians. Scientific sessions include Morphogenesis, Negenesis and Tissue Engineering; Genetics and Genodermatoses; Hair Structure (science and cosmetic); Stem Cells, Stem Cell Niches and Cicatricial Alopecia; Hormones, Hair Growth and Pattern Hair Loss; Comparative Biology and Transgenic Models; Immunobiology and Alopecia Areata; and Emerging Technologies and Therapies. The hair congress will end with a Free Papers session.

An added bonus for attendees will be the Annual Scientific Meeting of the Australasian Society For Dermatology Research, which follows immediately after the World Hair Research Congress on the 19^th^ and 20^th^ of June. Invited international speakers include Paul Bowden, George Cotsarelis, Takashi Hashimoto, Pritinder Kaur, John McGrath, Irwin McLean, Ralf Paus, Hiroshi Shimizu and John Sundberg.

In addition to the scientific events, we have organized a delightful social program in a beautiful environment. Cairns is the gateway to the Great Barrier Reef and other natural wonders. Cairns, being in Northern Australia (Queensland), is convenient for international travellers and has a wonderful climate. June is the best time of the year to visit Cairns-as they say in Queensland, "beautiful one day, perfect the next." The conference dinner will be held at *Rainforestation Nature Park* (http://www.rainforest.com.au/index.htm), where everyone will have a chance to cuddle a Koala, feed a Kangaroo, throw a boomerang and play the didgeridoo. If a visit to the Great Barrier Reef is on your list of things to do in your lifetime, there will never be a better time than June 16-19, 2010.

We would like to thank our sponsors in advance for their support, specifically Johnson and Johnson, Kao Corporation and the Australian Hair and Scalp Foundation. We are grateful for all the efforts by members of the local and international advisory boards and session chairs in promoting this event.

Abstract submissions are now open. Please visit www.hair2010.org for registration and abstract submission information.

